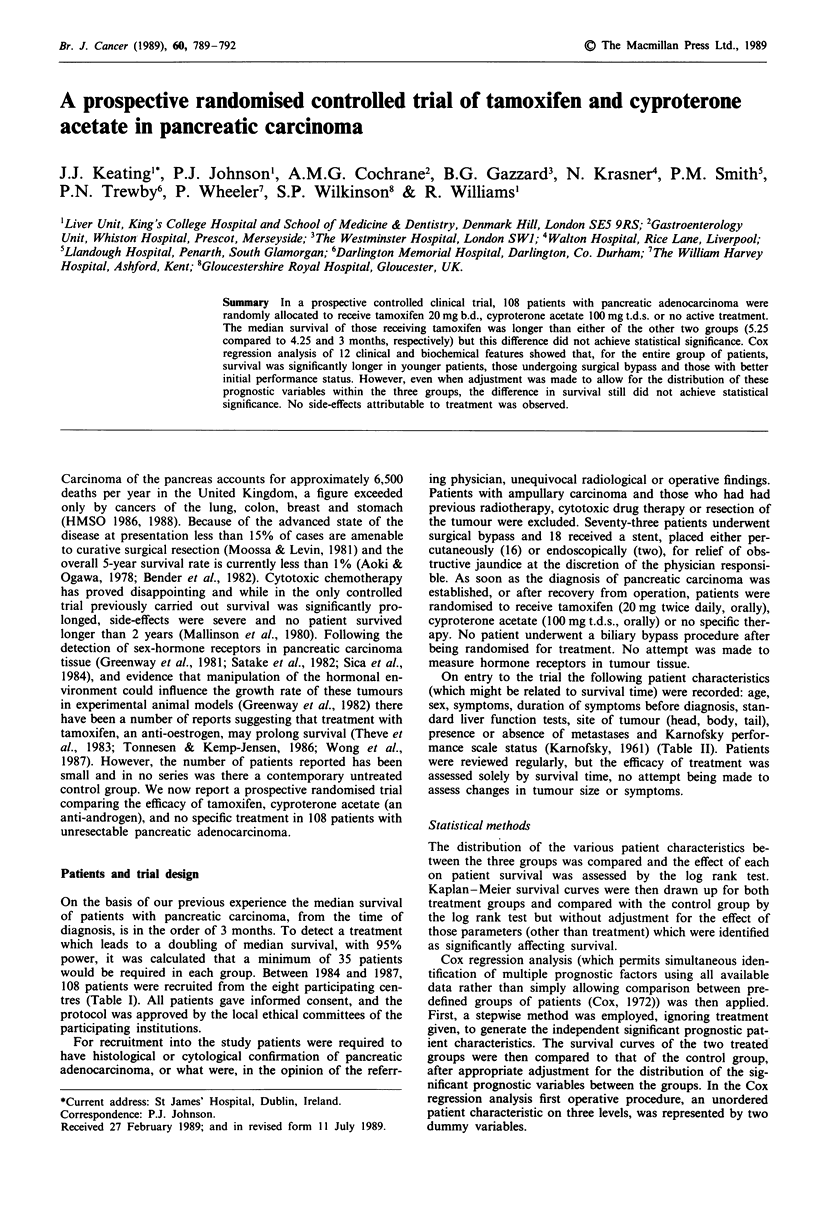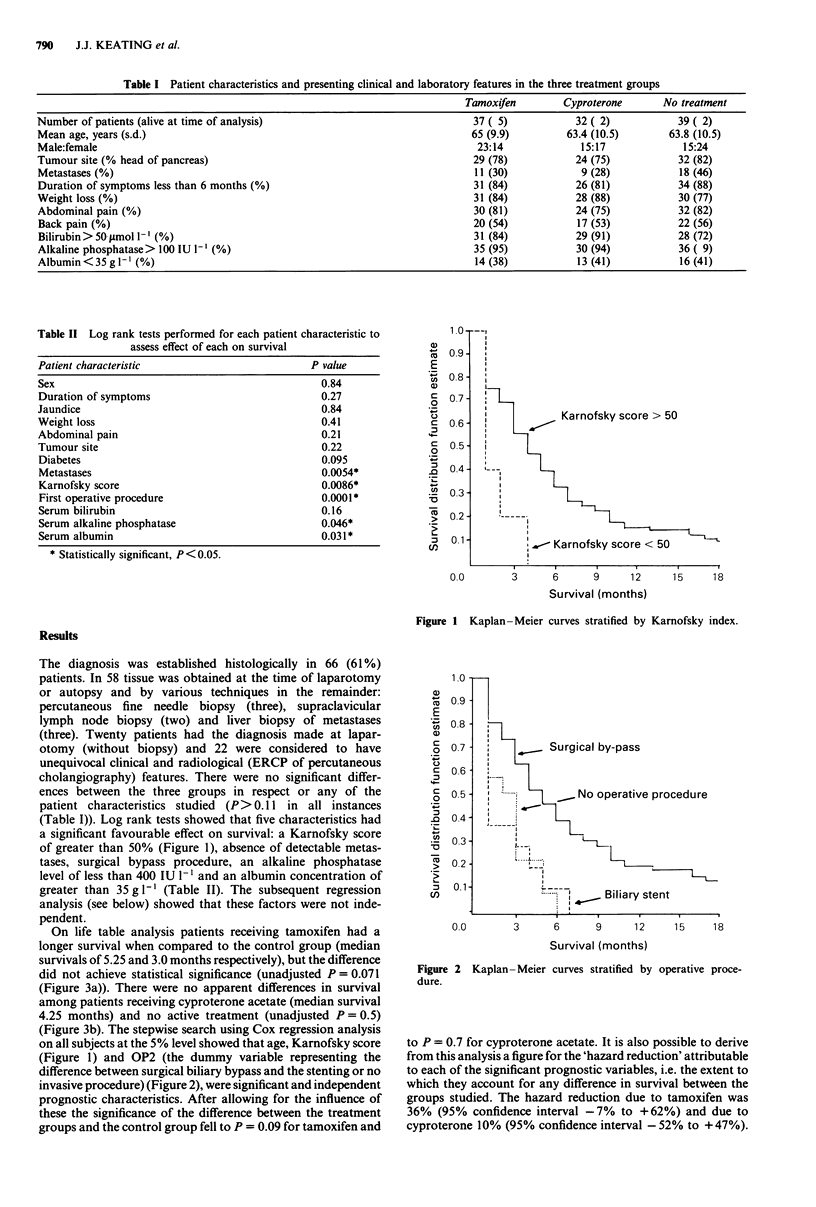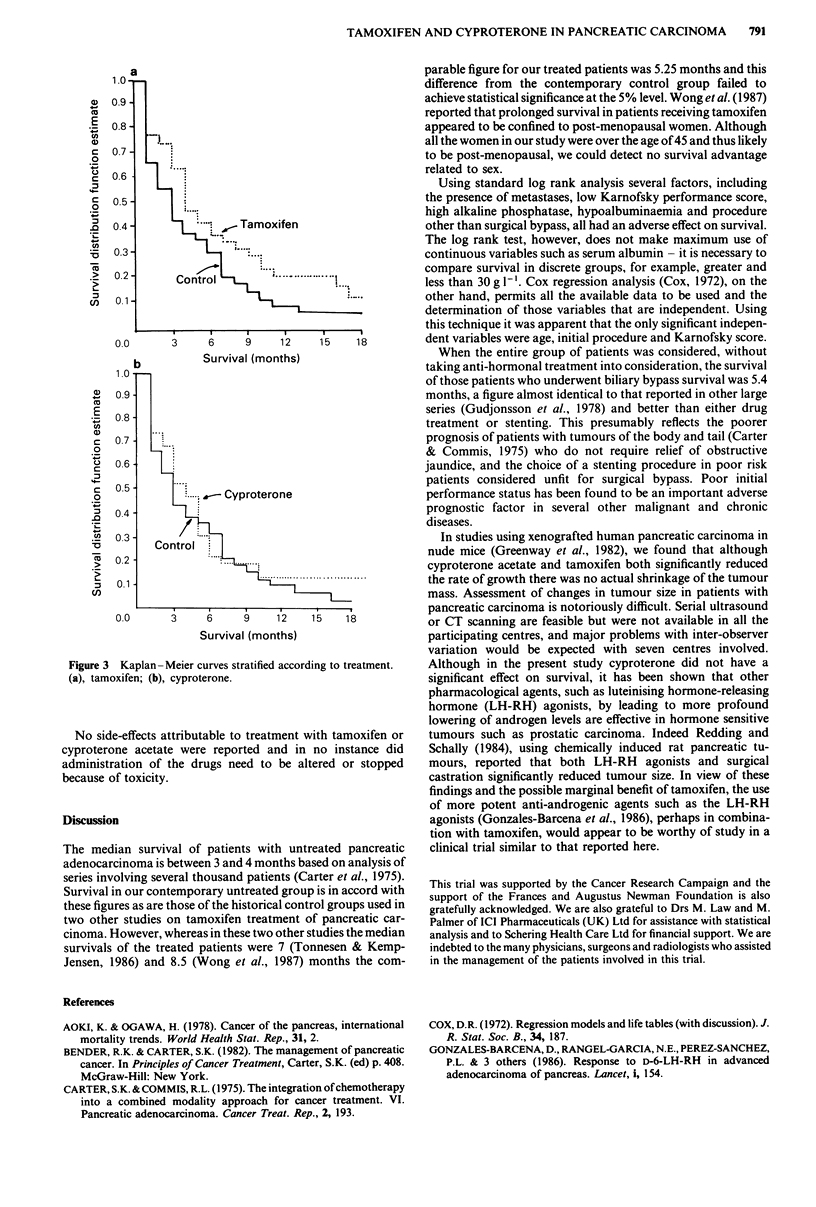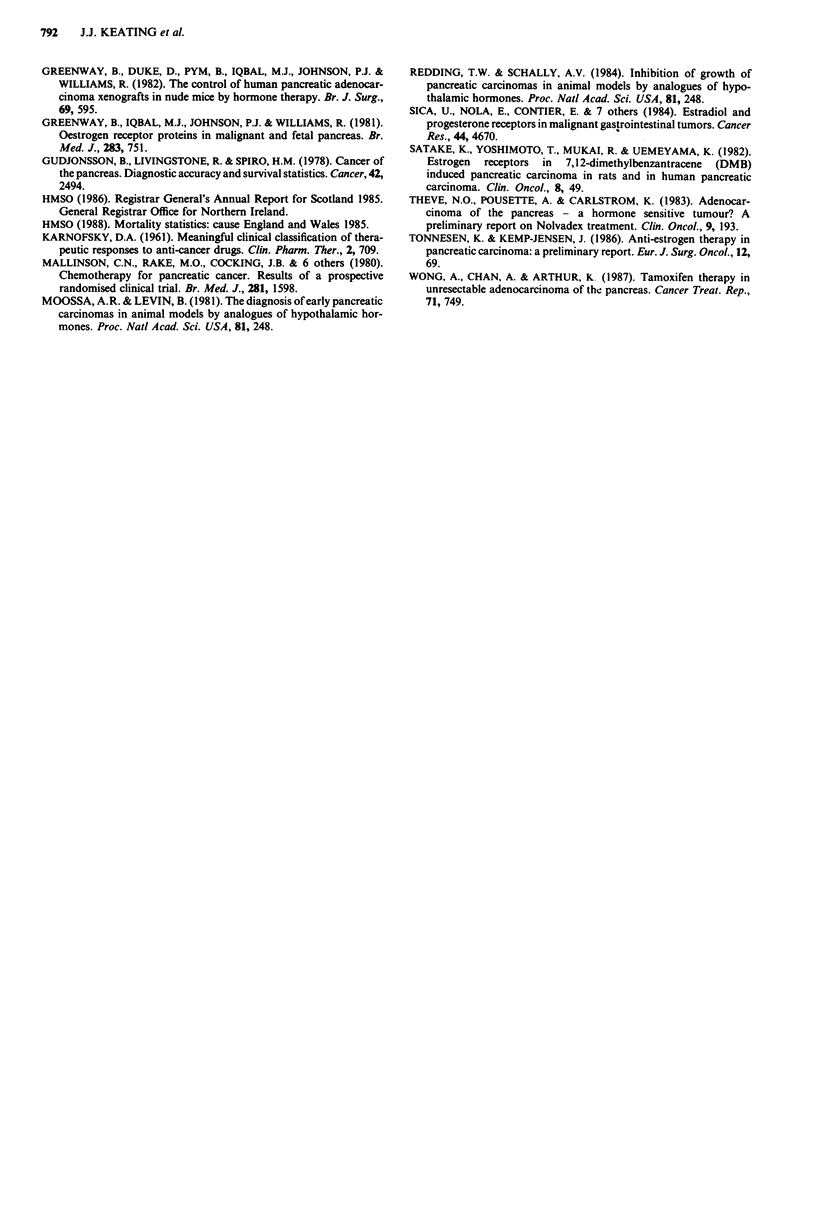# A prospective randomised controlled trial of tamoxifen and cyproterone acetate in pancreatic carcinoma.

**DOI:** 10.1038/bjc.1989.361

**Published:** 1989-11

**Authors:** J. J. Keating, P. J. Johnson, A. M. Cochrane, B. G. Gazzard, N. Krasner, P. M. Smith, P. N. Trewby, P. Wheeler, S. P. Wilkinson, R. Williams

**Affiliations:** Liver Unit, King's College Hospital, Denmark Hill, London, UK.

## Abstract

In a prospective controlled clinical trial, 108 patients with pancreatic adenocarcinoma were randomly allocated to receive tamoxifen 20 mg b.d., cyproteron acetate 100 mg t.d.s. or no active treatment. The median survival of those receiving tamoxifen was longer than either of the other two groups (5.25 compared to 4.25 and 3 months, respectively) but this difference did not achieve statistical significance. Cox regression analysis of 12 clinical and biochemical features showed that, for the entire group of patients, survival was significantly longer in younger patients, those undergoing surgical bypass and those with better initial performance status. However, even when adjustment was made to allow for the distribution of these prognostic variables within the three groups, the difference in survival still did not achieve statistical significance. No side-effects attributable to treatment was observed.


Br. J. Cancer (1989), 60, 789-792 © The Macmillan Press Ltd., 1989

### A prospective randomised controlled trial of tamoxifen and cyproterone acetate in pancreatic carcinoma

J.J. Keating[1*], P.J. Johnson[1], A.M.G. Cochrane[2], B.G. Gazzard[3], N. Krasner[4], P.M. Smith[5], P.N. Trewby[6], P. Wheeler[7], S.P. Wilkinson[8] & R. Williams[1]

[1]*Liver Unit, King's College Hospital and School of Medicine & Dentistry, Denmark Hill, London SE5 9RS;* [2]*Gastroenterology Unit, Whiston Hospital, Prescot, Merseyside;* [3]*The Westminster Hospital, London SW1;* [4]*Walton Hospital, Rice Lane, Liverpool;* [5]*Llandough Hospital, Penarth, South Glamorgan;* [6]*Darlington Memorial Hospital, Darlington, Co. Durham;* [7]*The William Harvey Hospital, Ashford, Kent;* [8]*Gloucestershire Royal Hospital, Gloucester, UK.*

**Summary** In a prospective controlled clinical trial, 108 patients with pancreatic adenocarcinoma were randomly allocated to receive tamoxifen 20 mg b.d., cyproterone acetate 100 mg t.d.s. or no active treatment. The median survival of those receiving tamoxifen was longer than either of the other two groups (5.25 compared to 4.25 and 3 months, respectively) but this difference did not achieve statistical significance. Cox regression analysis of 12 clinical and biochemical features showed that, for the entire group of patients, survival was significantly longer in younger patients, those undergoing surgical bypass and those with better initial performance status. However, even when adjustment was made to allow for the distribution of these prognostic variables within the three groups, the difference in survival still did not achieve statistical significance. No side-effects attributable to treatment was observed.

Carcinoma of the pancreas accounts for approximately 6,500 deaths per year in the United Kingdom, a figure exceeded only by cancers of the lung, colon, breast and stomach (HMSO 1986, 1988). Because of the advanced state of the disease at presentation less than 15% of cases are amenable to curative surgical resection (Moossa & Levin, 1981) and the overall 5-year survival rate is currently less than 1% (Aoki & Ogawa, 1978; Bender *et al.*, 1982). Cytotoxic chemotherapy has proved disappointing and while in the only controlled trial previously carried out survival was significantly prolonged, side-effects were severe and no patient survived longer than 2 years (Mallinson *et al.*, 1980). Following the detection of sex-hormone receptors in pancreatic carcinoma tissue (Greenway *et al.*, 1981; Satake *et al.*, 1982; Sica *et al.*, 1984), and evidence that manipulation of the hormonal environment could influence the growth rate of these tumours in experimental animal models (Greenway *et al.*, 1982) there have been a number of reports suggesting that treatment with tamoxifen, an anti-oestrogen, may prolong survival (Theve *et al.*, 1983; Tonnesen & Kemp-Jensen, 1986; Wong *et al.*, 1987). However, the number of patients reported has been small and in no series was there a contemporary untreated control group. We now report a prospective randomised trial comparing the efficacy of tamoxifen, cyproterone acetate (an anti-androgen), and no specific treatment in 108 patients with unresectable pancreatic adenocarcinoma.

### Patients and trial design

On the basis of our previous experience the median survival of patients with pancreatic carcinoma, from the time of diagnosis, is in the order of 3 months. To detect a treatment which leads to a doubling of median survival, with 95% power, it was calculated that a minimum of 35 patients would be required in each group. Between 1984 and 1987, 108 patients were recruited from the eight participating centres (Table I). All patients gave informed consent, and the protocol was approved by the local ethical committees of the participating institutions.

For recruitment into the study patients were required to have histological or cytological confirmation of pancreatic adenocarcinoma, or what were, in the opinion of the referring physician, unequivocal radiological or operative findings. Patients with ampullary carcinoma and those who had had previous radiotherapy, cytotoxic drug therapy or resection of the tumour were excluded. Seventy-three patients underwent surgical bypass and 18 received a stent, placed either percutaneously (16) or endoscopically (two), for relief of obstructive jaundice at the discretion of the physician responsible. As soon as the diagnosis of pancreatic carcinoma was established, or after recovery from operation, patients were randomised to receive tamoxifen (20 mg twice daily, orally), cyproterone acetate (100 mg t.d.s., orally) or no specific therapy. No patient underwent a biliary bypass procedure after being randomised for treatment. No attempt was made to measure hormone receptors in tumour tissue.

On entry to the trial the following patient characteristics (which might be related to survival time) were recorded: age, sex, symptoms, duration of symptoms before diagnosis, standard liver function tests, site of tumour (head, body, tail), presence or absence of metastases and Karnofsky performance scale status (Karnofsky, 1961) (Table II). Patients were reviewed regularly, but the efficacy of treatment was assessed solely by survival time, no attempt being made to assess changes in tumour size or symptoms.

### *Statistical methods*

The distribution of the various patient characteristics between the three groups was compared and the effect of each on patient survival was assessed by the log rank test. Kaplan-Meier survival curves were then drawn up for both treatment groups and compared with the control group by the log rank test but without adjustment for the effect of those parameters (other than treatment) which were identified as significantly affecting survival.

Cox regression analysis (which permits simultaneous identification of multiple prognostic factors using all available data rather than simply allowing comparison between predefined groups of patients (Cox, 1972)) was then applied. First, a stepwise method was employed, ignoring treatment given, to generate the independent significant prognostic patient characteristics. The survival curves of the two treated groups were then compared to that of the control group, after appropriate adjustment for the distribution of the significant prognostic variables between the groups. In the Cox regression analysis first operative procedure, an unordered patient characteristic on three levels, was represented by two dummy variables.

*Current address: St James' Hospital, Dublin, Ireland.
Correspondence: P.J. Johnson.
Received 27 February 1989; and in revised form 11 July 1989.

**Table I** Patient characteristics and presenting clinical and laboratory features in the three treatment groups

| | *Tamoxifen* | *Cyproterone* | *No treatment* |
|---|---|---|---|
| Number of patients (alive at time of analysis) | 37 ( 5) | 32 ( 2) | 39 ( 2) |
| Mean age, years (s.d.) | 65 (9.9) | 63.4 (10.5) | 63.8 (10.5) |
| Male:female | 23:14 | 15:17 | 15:24 |
| Tumour site (% head of pancreas) | 29 (78) | 24 (75) | 32 (82) |
| Metastases (%) | 11 (30) | 9 (28) | 18 (46) |
| Duration of symptoms less than 6 months (%) | 31 (84) | 26 (81) | 34 (88) |
| Weight loss (%) | 31 (84) | 28 (88) | 30 (77) |
| Abdominal pain (%) | 30 (81) | 24 (75) | 32 (82) |
| Back pain (%) | 20 (54) | 17 (53) | 22 (56) |
| Bilirubin > 50 $\mu$mol $l^{-1}$ (%) | 31 (84) | 29 (91) | 28 (72) |
| Alkaline phosphatase > 100 IU $l^{-1}$ (%) | 35 (95) | 30 (94) | 36 ( 9) |
| Albumin < 35 g $l^{-1}$ (%) | 14 (38) | 13 (41) | 16 (41) |

**Table II** Log rank tests performed for each patient characteristic to assess effect of each on survival

| *Patient characteristic* | *P value* |
|---|---|
| Sex | 0.84 |
| Duration of symptoms | 0.27 |
| Jaundice | 0.84 |
| Weight loss | 0.41 |
| Abdominal pain | 0.21 |
| Tumour site | 0.22 |
| Diabetes | 0.095 |
| Metastases | 0.0054* |
| Karnofsky score | 0.0086* |
| First operative procedure | 0.0001* |
| Serum bilirubin | 0.16 |
| Serum alkaline phosphatase | 0.046* |
| Serum albumin | 0.031* |

* Statistically significant, $P < 0.05$.

**Figure 1** Kaplan–Meier curves stratified by Karnofsky index.

### Results

The diagnosis was established histologically in 66 (61%) patients. In 58 tissue was obtained at the time of laparotomy or autopsy and by various techniques in the remainder: percutaneous fine needle biopsy (three), supraclavicular lymph node biopsy (two) and liver biopsy of metastases (three). Twenty patients had the diagnosis made at laparotomy (without biopsy) and 22 were considered to have unequivocal clinical and radiological (ERCP of percutaneous cholangiography) features. There were no significant differences between the three groups in respect or any of the patient characteristics studied ($P > 0.11$ in all instances (Table I)). Log rank tests showed that five characteristics had a significant favourable effect on survival: a Karnofsky score of greater than 50% (Figure 1), absence of detectable metastases, surgical bypass procedure, an alkaline phosphatase level of less than 400 IU $l^{-1}$ and an albumin concentration of greater than 35 g $l^{-1}$ (Table II). The subsequent regression analysis (see below) showed that these factors were not independent.

On life table analysis patients receiving tamoxifen had a longer survival when compared to the control group (median survivals of 5.25 and 3.0 months respectively), but the difference did not achieve statistical significance (unadjusted $P = 0.071$ (Figure 3a)). There were no apparent differences in survival among patients receiving cyproterone acetate (median survival 4.25 months) and no active treatment (unadjusted $P = 0.5$) (Figure 3b). The stepwise search using Cox regression analysis on all subjects at the 5% level showed that age, Karnofsky score (Figure 1) and OP2 (the dummy variable representing the difference between surgical biliary bypass and the stenting or no invasive procedure) (Figure 2), were significant and independent prognostic characteristics. After allowing for the influence of these the significance of the difference between the treatment groups and the control group fell to $P = 0.09$ for tamoxifen and

**Figure 2** Kaplan–Meier curves stratified by operative procedure.

to $P = 0.7$ for cyproterone acetate. It is also possible to derive from this analysis a figure for the 'hazard reduction' attributable to each of the significant prognostic variables, i.e. the extent to which they account for any difference in survival between the groups studied. The hazard reduction due to tamoxifen was 36% (95% confidence interval −7% to +62%) and due to cyproterone 10% (95% confidence interval −52% to +47%).

**Figure 3** Kaplan-Meier curves stratified according to treatment. (**a**), tamoxifen; (**b**), cyproterone.

No side-effects attributable to treatment with tamoxifen or cyproterone acetate were reported and in no instance did administration of the drugs need to be altered or stopped because of toxicity.

### Discussion

The median survival of patients with untreated pancreatic adenocarcinoma is between 3 and 4 months based on analysis of series involving several thousand patients (Carter *et al.*, 1975). Survival in our contemporary untreated group is in accord with these figures as are those of the historical control groups used in two other studies on tamoxifen treatment of pancreatic carcinoma. However, whereas in these two other studies the median survivals of the treated patients were 7 (Tonnesen & Kemp-Jensen, 1986) and 8.5 (Wong *et al.*, 1987) months the comparable figure for our treated patients was 5.25 months and this difference from the contemporary control group failed to achieve statistical significance at the 5% level. Wong *et al.* (1987) reported that prolonged survival in patients receiving tamoxifen appeared to be confined to post-menopausal women. Although all the women in our study were over the age of 45 and thus likely to be post-menopausal, we could detect no survival advantage related to sex.

Using standard log rank analysis several factors, including the presence of metastases, low Karnofsky performance score, high alkaline phosphatase, hypoalbuminaemia and procedure other than surgical bypass, all had an adverse effect on survival. The log rank test, however, does not make maximum use of continuous variables such as serum albumin – it is necessary to compare survival in discrete groups, for example, greater and less than 30 $g\,l^{-1}$. Cox regression analysis (Cox, 1972), on the other hand, permits all the available data to be used and the determination of those variables that are independent. Using this technique it was apparent that the only significant independent variables were age, initial procedure and Karnofsky score.

When the entire group of patients was considered, without taking anti-hormonal treatment into consideration, the survival of those patients who underwent biliary bypass survival was 5.4 months, a figure almost identical to that reported in other large series (Gudjonsson *et al.*, 1978) and better than either drug treatment or stenting. This presumably reflects the poorer prognosis of patients with tumours of the body and tail (Carter & Commis, 1975) who do not require relief of obstructive jaundice, and the choice of a stenting procedure in poor risk patients considered unfit for surgical bypass. Poor initial performance status has been found to be an important adverse prognostic factor in several other malignant and chronic diseases.

In studies using xenografted human pancreatic carcinoma in nude mice (Greenway *et al.*, 1982), we found that although cyproterone acetate and tamoxifen both significantly reduced the rate of growth there was no actual shrinkage of the tumour mass. Assessment of changes in tumour size in patients with pancreatic carcinoma is notoriously difficult. Serial ultrasound or CT scanning are feasible but were not available in all the participating centres, and major problems with inter-observer variation would be expected with seven centres involved. Although in the present study cyproterone did not have a significant effect on survival, it has been shown that other pharmacological agents, such as luteinising hormone-releasing hormone (LH-RH) agonists, by leading to more profound lowering of androgen levels are effective in hormone sensitive tumours such as prostatic carcinoma. Indeed Redding and Schally (1984), using chemically induced rat pancreatic tumours, reported that both LH-RH agonists and surgical castration significantly reduced tumour size. In view of these findings and the possible marginal benefit of tamoxifen, the use of more potent anti-androgenic agents such as the LH-RH agonists (Gonzales-Barcena *et al.*, 1986), perhaps in combination with tamoxifen, would appear to be worthy of study in a clinical trial similar to that reported here.

This trial was supported by the Cancer Research Campaign and the support of the Frances and Augustus Newman Foundation is also gratefully acknowledged. We are also grateful to Drs M. Law and M. Palmer of ICI Pharmaceuticals (UK) Ltd for assistance with statistical analysis and to Schering Health Care Ltd for financial support. We are indebted to the many physicians, surgeons and radiologists who assisted in the management of the patients involved in this trial.

### References

AOKI, K. & OGAWA, H. (1978). Cancer of the pancreas, international mortality trends. *World Health Stat. Rep.*, **31**, 2.

BENDER, R.K. & CARTER, S.K. (1982). The management of pancreatic cancer. In *Principles of Cancer Treatment*, Carter, S.K. (ed) p. 408. McGraw-Hill: New York.

CARTER, S.K. & COMMIS, R.L. (1975). The integration of chemotherapy into a combined modality approach for cancer treatment. VI. Pancreatic adenocarcinoma. *Cancer Treat. Rep.*, **2**, 193.

COX, D.R. (1972). Regression models and life tables (with discussion). *J. R. Stat. Soc. B.*, **34**, 187.

GONZALES-BARCENA, D., RANGEL-GARCIA, N.E., PEREZ-SANCHEZ, P.L. & 3 others (1986). Response to D-6-LH-RH in advanced adenocarcinoma of pancreas. *Lancet*, **i**, 154.

GREENWAY, B., DUKE, D., PYM, B., IQBAL, M.J., JOHNSON, P.J. & WILLIAMS, R. (1982). The control of human pancreatic adenocarcinoma xenografts in nude mice by hormone therapy. *Br. J. Surg.*, **69**, 595.

GREENWAY, B., IQBAL, M.J., JOHNSON, P.J. & WILLIAMS, R. (1981). Oestrogen receptor proteins in malignant and fetal pancreas. *Br. Med. J.*, **283**, 751.

GUDJONSSON, B., LIVINGSTONE, R. & SPIRO, H.M. (1978). Cancer of the pancreas. Diagnostic accuracy and survival statistics. *Cancer*, **42**, 2494.

HMSO (1986). Registrar General's Annual Report for Scotland 1985. General Registrar Office for Northern Ireland.

HMSO (1988). Mortality statistics: cause England and Wales 1985.

KARNOFSKY, D.A. (1961). Meaningful clinical classification of therapeutic responses to anti-cancer drugs. *Clin. Pharm. Ther.*, **2**, 709.

MALLINSON, C.N., RAKE, M.O., COCKING, J.B. & 6 others (1980). Chemotherapy for pancreatic cancer. Results of a prospective randomised clinical trial. *Br. Med. J.*, **281**, 1598.

MOOSSA, A.R. & LEVIN, B. (1981). The diagnosis of early pancreatic carcinomas in animal models by analogues of hypothalamic hormones. *Proc. Natl Acad. Sci. USA*, **81**, 248.

REDDING, T.W. & SCHALLY, A.V. (1984). Inhibition of growth of pancreatic carcinomas in animal models by analogues of hypothalamic hormones. *Proc. Natl Acad. Sci. USA*, **81**, 248.

SICA, U., NOLA, E., CONTIER, E. & 7 others (1984). Estradiol and progesterone receptors in malignant gastrointestinal tumors. *Cancer Res.*, **44**, 4670.

SATAKE, K., YOSHIMOTO, T., MUKAI, R. & UEMEYAMA, K. (1982). Estrogen receptors in 7,12-dimethylbenzantracene (DMB) induced pancreatic carcinoma in rats and in human pancreatic carcinoma. *Clin. Oncol.*, **8**, 49.

THEVE, N.O., POUSETTE, A. & CARLSTROM, K. (1983). Adenocarcinoma of the pancreas – a hormone sensitive tumour? A preliminary report on Nolvadex treatment. *Clin. Oncol.*, **9**, 193.

TONNESEN, K. & KEMP-JENSEN, J. (1986). Anti-estrogen therapy in pancreatic carcinoma: a preliminary report. *Eur. J. Surg. Oncol.*, **12**, 69.

WONG, A., CHAN, A. & ARTHUR, K. (1987). Tamoxifen therapy in unresectable adenocarcinoma of the pancreas. *Cancer Treat. Rep.*, **71**, 749.